# Valence conversion and site reconstruction in near-infrared-emitting chromium-activated garnet for simultaneous enhancement of quantum efficiency and thermal stability

**DOI:** 10.1038/s41377-023-01283-3

**Published:** 2023-10-07

**Authors:** Dongjie Liu, Guogang Li, Peipei Dang, Qianqian Zhang, Yi Wei, Lei Qiu, Hongzhou Lian, Mengmeng Shang, Jun Lin

**Affiliations:** 1 State Key Laboratory of Rare Earth Resource Utilization, Changchun Institute of Applied Chemistry, Chinese Academy of Sciences, Changchun, 130022 China; 2 Faculty of Materials Science and Chemistry, China University of Geosciences, Wuhan, 430074 China; 3 Zhejiang Institute, China University of Geosciences, Hangzhou, 311305 China; 4 University of Science and Technology of China, Hefei, 230026 China; 5 School of Material Science and Engineering, Shandong University, Jinan, 266071 China

**Keywords:** Optical properties of diamond, Inorganic LEDs

## Abstract

Achievement of high photoluminescence quantum efficiency and thermal stability is challenging for near-infrared (NIR)-emitting phosphors. Here, we designed a “kill two birds with one stone” strategy to simultaneously improve quantum efficiency and thermal stability of the NIR-emitting Ca_3_Y_2-2*x*_(ZnZr)_*x*_Ge_3_O_12_:Cr garnet system by chemical unit cosubstitution, and revealed universal structure-property relationship and the luminescence optimization mechanism. The cosubstitution of [Zn^2+^–Zr^4+^] for [Y^3+^–Y^3+^] played a critical role as reductant to promote the valence transformation from Cr^4+^ to Cr^3+^, resulting from the reconstruction of octahedral sites for Cr^3+^. The introduction of [Zn^2+^–Zr^4+^] unit also contributed to a rigid crystal structure. These two aspects together realized the high internal quantum efficiency of 96% and excellent thermal stability of 89%@423 K. Moreover, information encryption with “burning after reading” was achieved based on different chemical resistance of the phosphors to acid. The developed NIR-emitting phosphor-converted light-emitting diode demonstrated promising applications in bio-tissue imaging and night vision. This work provides a new perspective for developing high-performance NIR-emitting phosphor materials.

## Abstract (web summary)

Significant improvement of quantum efficiency and thermal stability of NIR-emitting Ca_3_Y_2-2*x*_(ZnZr)_*x*_Ge_3_O_12_:Cr was achieved by chemical unit cosubstitution, benefiting from valence reduction of Cr^4+^ to Cr^3+^ and reconstructed rigid crystal structure.

## Graphical abstract

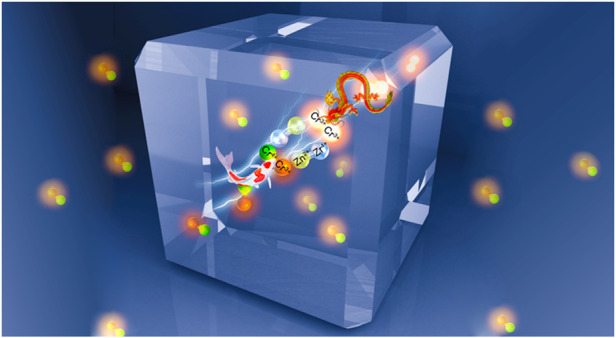

## Introduction

Near-infrared (NIR) luminescent materials have attracted wide attention in emerging technology fields such as NIR spectroscopic analysis, bioimaging, and night vision^1–6^. To meet the current demands of portable NIR light sources, NIR-emitting phosphor-converted light-emitting diodes (pc-LEDs) have been widely developed due to their compactness and tunable broadband emission^7–10^. The key is to exploit blue-light excitable high-performance NIR-emitting phosphor materials. Numerous NIR-emitting phosphors have been discovered by doping rare-earth and transition metal ions, among which Cr^3+^-activated phosphors stand out given that they can easily generate broadband NIR emission in the range from 700 to 1100 nm^11–16^. This enables their applications in smart devices that are integrated with Si-based detectors^17^. Recently, progress has been made in realizing tunable broadband emission of Cr^3+^-activated phosphors^18–20^. However, the major issues still remain their unsatisfactory photoluminescence quantum efficiency and poor thermal quenching resistance, restricting their practical applications in pc-LEDs.

To address these issues, researchers have focused on the Cr^3+^-activated garnet-structured NIR-emitting phosphors^21–25^. The ideal garnets are assigned to the *Ia*-3*d* space group of cubic crystal systems with a chemical formula of A_3_B_2_C_3_O_12_. There are dodecahedra (A), octahedra (B), and tetrahedra (C) in garnet structures. The dodecahedra and octahedra are connected through edge-sharing, while the octahedra and tetrahedra are connected by vertex-sharing. The diversity of cation selection in garnets results in a great number of chemical variants. A_3_B_2_C_3_O_12_-typed garnets are considered promising host materials that can provide Cr^3+^ ions with octahedral B sites, compact coordinated environment, and tunable structures, and thereby diverse luminescence properties including the required ones can be expected^26–29^. For example, Gd_3_Zn_*x*_Ga_5-2*x*_Ge_*x*_O_12_:Cr^3+^ show tunable broadband NIR emission via crystal field engineering; while Y_3_(Al,Mg)_2_(Al,Si)_3_O_12_:Cr^3+^ exhibit efficient and thermal quenching resistant NIR emission^30,31^. Unfortunately, there is a trade-off between the emission wavelength and quantum efficiency as well as thermal stability. That is, the highly efficient and thermally stable NIR luminescence is generally accompanied by short emission wavelength (<750 nm), which limits the applications of these garnet phosphors in some scenarios like food analysis^32,33^. Therefore, how to improve luminescence efficiency and thermal stability while maintaining wavelength within specific range is still a challenge for Cr^3+^-doped NIR-emitting garnet phosphors.

In this regard, two dominant factors affecting the luminescence of Cr^3+^-doped garnet phosphors should be mentioned. One is the luminescence “killer” Cr^4+^ that shows intensive absorption in NIR region^34^. The common high-temperature synthesis of the phosphors in air and the existed tetrahedral C sites in A_3_B_2_C_3_O_12_ garnets synergistically favor the formation of Cr^4+^ ions^35,36^. Although it can be partly prevented by reducing atmosphere, this way is not suitable for all host materials^25^. But from another perspective, this problem can be solved by

constructing more appropriate crystallographic B sites for accommodating Cr^3+^ ions to increase their formation competitiveness in phosphors. Another crucial factor is structural rigidity. A rigid host structure can weaken the electron-phonon coupling effect and suppress the nonradiative transition^20,22^.

In this work, the underlying luminescence optimization mechanism of Ca_3_Y_2-2*x*_(ZnZr)_*x*_Ge_3_O_12_:Cr garnet system was investigated based on structural analysis and density functional theory (DFT) calculations. The coexistence of Cr^3+^ and Cr^4+^ in Ca_3_Y_2_Ge_3_O_12_:Cr was demonstrated to be responsible for its low quantum efficiency. With reconstruction of the crystallographic B sites for Cr^3+^ ions in Ca_3_Y_2_Ge_3_O_12_ by the designed cosubstitution of [Zn^2+^–Zr^4+^] for [Y^3+^–Y^3+^], Cr^4+^ luminescence killers were transformed to beneficial Cr^3+^ emission centers. The strategy of chemical unit cosubstitution killed two birds with one stone, which also successfully built a rigid crystal structure. These changes led to the significant improvement of photoluminescence quantum efficiency and thermal stability with only slight emission shift. Finally, information encryption inspired by chemical resistance difference was designed to achieve “burning after reading”. The great potential of the developed portable pc-LED in bio-tissue imaging and night-vision applications was also demonstrated.

## Results

### Crystal structure and phase identification

**Fig. 1:**
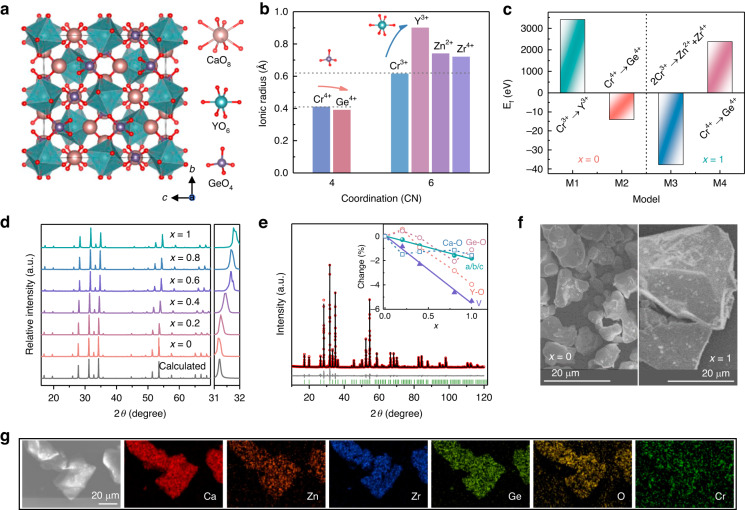
Structure and morphology characterizations of Ca_3_Y_2-2*x*_(ZnZr)_*x*_Ge_3_O_12_**:Cr (*x*= 0–1).** **a** Crystal structure of Ca_3_Y_2_Ge_3_O_12_. **b** Ionic radii for cation ions under corresponding coordination numbers. **c** Formation energy (E_f_) for different substitution models of Cr in Ca_3_Y_2_Ge_3_O_12_ and Ca_3_ZnZrGe_3_O_12_. **d** XRD patterns of Ca_3_Y_2-2*x*_(ZnZr)_*x*_Ge_3_O_12_:Cr (*x* = 0–1). **e** XRD Rietveld refinement of *x* = 1. The inset shows the relative changes in the cell parameters (*a*, *b*, and *c*), volumes (*V*), and bond lengths (Ca–O, Y/Zn/Zr–O, and Ge–O) with *x*. **f** SEM images of *x* = 0 and *x* = 1. **g** Element mapping images of Ca, Zn, Zr, Ge, O, and Cr elements in *x* = 1

Figure 1a shows the crystal structure of Ca_3_Y_2_Ge_3_O_12_, which adopts a typical garnet structure with formula A_3_B_2_C_3_O_12_. Ca^2+^, Y^3+^, and Ge^4+^ ions are distributed at the dodecahedral A sites, octahedral B sites, and tetrahedral C sites, respectively. Considering that the crystal field stabilization energy of Cr^3+^ in octahedral site is eight times higher than that in tetrahedral site, Cr^3+^ ions preferentially occupy the octahedral Y^3+^ sites in Ca_3_Y_2_Ge_3_O_12_ host, while maintaining charge balance^37,38^. However, such a substitution can cause large lattice strain due to the obvious size-mismatch between Cr^3+^ and Y^3+^ ions (Fig. 1b and Table S1). This increases the substitution difficulty, which can be evaluated by the parameter *D*_r_ obtained from the empirical formula^39^:

1 $${D}_{r}=\frac{\left|{R}_{r}-{R}_{d}\right|}{{R}_{r}}\times 100 \%$$

where *D*_r_ represents the difference of ion radii between the doped ions and host cations, *R*_r_ and *R*_d_ are the radii of the host cations and the doped ions, respectively. The substitution process is believed to be possible on condition that the *D*_r_ is less than 30%. *D*_r_ for Cr^3+^ substituting Y^3+^ is 31.7%, which means the substitution is difficult. That is, it is unfavorable for Cr^3+^ ions to replace Y^3+^ ions. However, *D*_r_ for Cr^4+^ substituting Ge^4+^ is only 5.1%, indicating that chromium ions are very likely to enter the Ge^4+^ sites in the form of tetravalent Cr^4+^ ions. To maintain trivalent state of chromium ions in the host, octahedral sites of appropriate size should be constructed to accommodate Cr^3+^. Smaller Zn^2+^ and Zr^4+^ ions were introduced into octahedral Y sites by the designed chemical unit cosubstitution of [Zn^2+^–Zr^4+^] for [Y^3+^–Y^3+^], which was expected to reduce the size-mismatch between Cr^3+^ ions and Y^3+^ sites so as to promote the formation of Cr^3+^ instead of Cr^4+^. The valence preferences of chromium ions in Ca_3_Y_2_Ge_3_O_12_ and Ca_3_ZnZrGe_3_O_12_ were simulated by DFT calculations. According to the above analysis, two substitution models for the two hosts were considered, respectively. That is, one Cr^3+^ ion occupied one Y^3+^ site (Model 1) and one Cr^4+^ ions occupied one Ge^4+^ site (Model 2) in Ca_3_Y_2_Ge_3_O_12_; two Cr^3+^ ions occupied one Zn^2+^ site and one Zr^4+^ site (Model 3), and one Cr^4+^ ions occupied one Ge^4+^ site (Model 4) in Ca_3_ZnZrGe_3_O_12_. As show in Fig. 1c, the formation energy values of Model 2 and Model 3 are significantly smaller than those of Model 1 and Model 4, respectively, which indicates that chromium ions tend to exist in the form of tetravalent Cr^4+^ ions in Ca_3_Y_2_Ge_3_O_12_ and trivalent Cr^3+^ ions in Ca_3_ZnZrGe_3_O_12_.

Figure 1d shows that the X-ray diffraction (XRD) patterns of Ca_3_Y_2-2*x*_(ZnZr)_*x*_Ge_3_O_12_:Cr (*x* = 0–1) are consistent with the calculated pattern of Ca_3_Y_2_Ge_3_O_12_, demonstrating the successful realization of cosubstitution. In addition, the XRD peak shifts toward higher angles, which is in accordance with the fact that Zn^2+^ and Zr^4+^ ions are smaller than Y^3+^ ions. Rietveld refinements (Fig. 1e and Fig. S1) were conducted to identify the pure phase and reveal the crystal structure changes in depth. The standard pattern of Ca_3_Y_2_Ge_3_O_12_ used for refinements in this work is Crystallography Open Database (COD) card no. 2009001. The refined results are listed in Tables S2–S3. As shown in the inset of Fig. 1e, linear changes of lattice parameters (*a*, *b*, and *c*) and cell volume (*V*) with *x* demonstrate that Ca_3_Y_2-2*x*_(ZnZr)_*x*_Ge_3_O_12_:Cr solid solutions are successfully synthesized. The lattice shows shrinkage with increasing *x*, which corresponds to the shift of XRD peak towards higher angles. In addition, it can be seen that the Y/Zn/Zr–O bonds shorten with a more obvious change rate as *x* increases from 0 to 1. This is ascribed to the cosubstitution of smaller [Zn^2+^–Zr^4+^] for [Y^3+^–Y^3+^] in conjunction with the transformation from Cr^4+^ to Cr^3+^. More smaller Cr^3+^ ions enter in larger Y/Zn/Zr sites can further decrease the Y/Zn/Zr–O bond lengths. As for the Ca–O bonds, they show less significant shortening. This is ascribed to the large volume of [CaO_8_] dodecahedra, which is less affected by the cosubstitution process. While Ge–O bonds can be easily affected by Y/Zn/Zr–O bonds due to the much smaller size of [GeO_4_] tetrahedra. The Ge–O bonds also exhibit less shortening to reduce the lattice strain caused by the shrinkage of [CaO_8_] dodecahedra and [Y/Zn/ZrO_6_] octahedra. Figure 1f and Fig. S2 show the irregular morphologies of *x* = 0 and *x* = 1 phosphors. It is noted that *x* = 1 shows bulk crystals much larger than *x* = 0, which is conducive to luminescence. The elemental mapping images of *x* = 1 are given in Fig. 1g, confirming the homogeneous distribution of Ca, Zn, Zr, Ge, Cr, and O elements.

### Photoluminescence properties

**Fig. 2:**
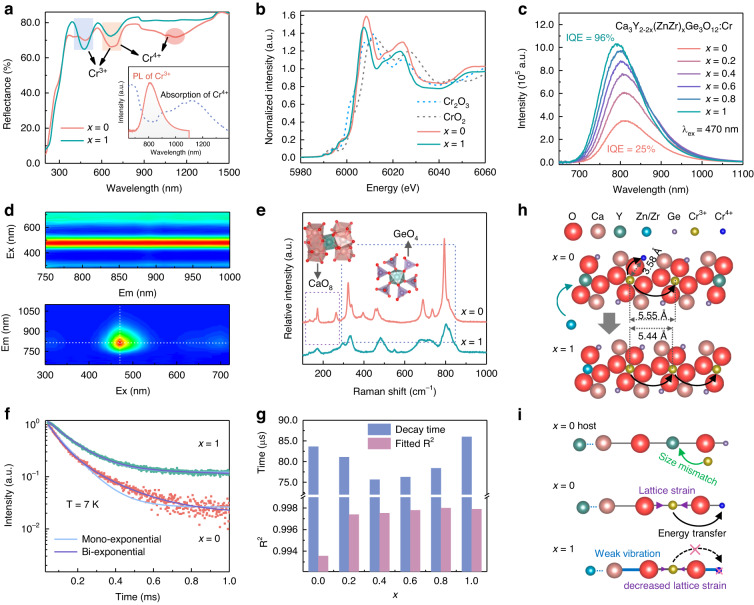
Photoluminescence properties of Ca_3_Y_2__-2*x*_(ZnZr)_*x*_Ge_3_O_12_**:Cr (*x*= 0–1).** **a** Diffuse reflectance spectra of *x* = 0 and *x* = 1. The inset shows the absorption spectrum of Cr^4+^ and emission spectrum of Cr^3+^ in *x* = 0. **b** Normalized Cr K-edge XANES spectra of Ca_3_Y_2-2*x*_(ZnZr)_*x*_Ge_3_O_12_:0.2Cr (*x* = 0 and 1) and reference compounds Cr_2_O_3_ and CrO_2_. **c** PL spectra of Ca_3_Y_2-2*x*_(ZnZr)_*x*_Ge_3_O_12_:Cr (*x* = 0–1). **d** Contour maps of normalized PLE spectra and PL spectra of *x* = 0 measured under different monitoring and excitation wavelengths, respectively. **e** Raman spectra of *x* = 0 and *x* = 1. **f** PL decay curves of *x* = 0 and *x* = 1 measured under 7 K, monitoring emission at 805 nm for *x* = 0 and 785 nm for *x* = 1. The excitation wavelength is 470 nm. **g** Lifetimes and fitted R^2^ values of Ca_3_Y_2-2*x*_(ZnZr)_*x*_Ge_3_O_12_:Cr (*x* = 0–1). **h** Schematic ion distribution in *x* = 0 and *x* = 1. The black arrows represent energy transfer. **i** Local structure change in *x* = 0 and *x* = 1. The large and small purple arrows represent the large and small lattice strain caused by doping, respectively. The thick blue lines represents the rigid bond with weak vibration. The black arrows represent energy transfer

The optimal doping concentration of Cr in Ca_3_Y_2_Ge_3_O_12_ host was determined to be 0.01 (Fig. S3). Thereafter, the luminescence properties of Ca_3_Y_2-2*x*_(ZnZr)_*x*_Ge_3_O_12_:Cr (*x* = 0–1) were regulated by the designed cosubstitution of [Zn^2+^–Zr^4+^] for [Y^3+^–Y^3+^]. As shown by the diffuse reflectance (DR) spectra in Fig. 2a, both *x* = 0 and *x* = 1 exhibit typical absorption peaks of Cr^3+^ at about 470 and 660 nm. However, Cr^4+^ ions are also observed in *x* = 0 noting an additional absorption signal at 1100 nm, which nearly disappears in *x* = 1. Moreover, the absorption peak of Cr^3+^ at 470 nm for *x* = 1 increases compared with *x* = 0, indicating the increased Cr^3+^ concentration in *x* = 1. The Cr K-edge X-ray absorption near-edge structure (XANES) spectra were measured to further determine the valence state of chromium. As shown in Fig. 2b, the main edge peaks of *x* = 0 (6007.4 eV) and *x* = 1 (6008.5 eV) are close to that of the standard Cr_2_O_3_, which suggests that chromium elements predominantly present the +3 oxidation state in the phosphors. However, it can be observed that the absorption edges of *x* = 0 and *x* = 1 are located between the absorption step of Cr_2_O_3_ and CrO_2_, which suggests the presence of both trivalent Cr^3+^ and tetravalent Cr^4+^ in the phosphors. Moreover, compared to *x* = 1, the absorption edge of *x* = 0 is closer to the absorption edge of CrO_2_, which means that there are more tetravalent Cr^4+^ ions in *x* = 0 than in *x* = 1. This result is consistent with the result shown by DRS. These results suggest the reduction of Cr^4+^ to Cr^3+^ with *x* increasing from 0 to 1, which supports the assumption that the cosubstitution of [Zn^2+^–Zr^4+^] for [Y^3+^–Y^3+^] contributes to the formation of trivalent Cr^3+^ ions. As the absorption spectrum of Cr^4+^ partially overlaps the emission spectrum of *x* = 0 (inset of Fig. 2a), the existence of Cr^4+^ can certainly reduce the photoluminescence quantum efficiency of Cr^3+^. The photoluminescence excitation (PLE) spectra of Ca_3_Y_2-2*x*_(ZnZr)_*x*_Ge_3_O_12_:Cr (*x* = 0–1) phosphors in Fig. S4a correspond to those absorption peaks in DR spectra, which shows optimal absorption and excitation in the blue-light region, matching with commercial blue LED chips. The photoluminescence (PL) spectra of Ca_3_Y_2-2*x*_(ZnZr)_*x*_Ge_3_O_12_:Cr (*x* = 0–1) phosphors excited under 470 nm are shown in Fig. 2c, exhibiting broadband emission originating from ^4^T_2g_→^4^A_2g_ transition in the range of 650–1100 nm. As *x* increases from 0 to 1, the PL intensity is increased by 2.7 times accompanied by a slight blue shift of the emission peak from 812 to 795 nm (Fig. S4b, c). The transformation from Cr^4+^ to Cr^3+^ certainly plays a positive role in the luminescence enhancement. The blue shift of d–d transition for Cr^3+^ was understood by the spectroscopic parameters *D*_q_ and *B*. Fig. S5 shows that *D*_q_*/B* value increases with increasing *x*, which indicates the strengthened crystal field, as interpreted by Tanabe–Sugano diagram (details are provided in the Supporting Information, Fig. S6 and Table S4). Thus, the PL spectra show reasonable blue shift. Figure 2d shows the contour maps of the normalized PLE spectra and PL spectra of *x* = 0 measured under different conditions. No visible change in spectral profile and peak position in the PLE and PL spectra proves only one Cr^3+^ luminescent center in *x* = 0 phosphor. As shown in Fig. S7a, the PL spectrum of *x* = 0 phosphor measured under 7 K shows an almost symmetric emission peak at 805 nm without any new emission peaks appearing. Moreover, it can be seen that the two PLE spectra in Fig. S7b monitored at 750 and 880 nm completely overlap. These low-temperature results further demonstrate one luminescent center in *x* = 0, which is believed to be Cr^3+^ substituting Y^3+^ in the Ca_3_Y_2_Ge_3_O_12_ host.

To further explore the influence of the designed cosubstitution of on the local structures and PL properties, Raman spectra of *x* = 0 and *x* = 1 are compared in Fig. 2e. These Raman peaks at 100−280 and 280−1000 cm^−1^ are assigned to the [CaO_8_] and [GeO_4_] sites, respectively (Table S5)^40^. Apparently, the intensity of these Raman signals becomes much lower after the cosubstitution of [Zn^2+^–Zr^4+^] for [Y^3+^–Y^3+^], which means weaker vibrations of Ca–O and Ge–O bonds in *x* = 1^41^. In addition, the vibration peaks broaden greatly for *x* = 1, indicating changes in crystal environment. CaO_8_ and GeO_4_ are surrounded by YO_6_ in *x* = 0, while surrounded by ZnO_6_ and ZrO_6_ in *x* = 1. The more uneven environment surrounding CaO_8_ and GeO_4_ in *x* = 1 can lead to broader Raman peaks. It should be noted that Ca^2+^ and Ge^4+^ ions construct the second coordination shell of Cr^3+^ ions. As displayed in the inset of Fig. 2e, one [CrO_6_] octahedra is edge-shared with six [CaO_8_] dodecahedra and vertex-shared with six [GeO_4_] tetrahedra. On the one hand, the shorter Cr–O bonds caused by cosubstitution of [Zn^2+^–Zr^4+^] for [Y^3+^–Y^3+^] indicate more strengthening bond force between Cr^3+^ and O^2-^ ions, enabling a more rigid [CrO_6_] octahedra. On the other hand, weaker vibrations of the second coordination shells means that they have less influence on the vibrations of [CrO_6_] octahedra and offer a more rigid secondary coordination environment for Cr^3+^. The Debye temperature (*Θ*_D_) is regarded as an indicator of structural rigidity, which was theoretically calculated to be 495 K for Ca_3_Y_2_Ge_3_O_12_ and 564 K for Ca_3_ZnZrGe_3_O_12_^42,43^. The higher *Θ*_D_ of Ca_3_ZnZrGe_3_O_12_ demonstrates the fact that it provides a more rigid structure for Cr^3+^. This is conducive to its radiative recombination, consistent with the enhanced NIR emission.

The luminescence decay behaviors of *x* = 0 and *x* = 1 phosphors under 7 K were studied (Fig. 2f). The decay curves were measured under such low temperature, which minimize the influence of temperature on luminescence decay and can better reflect the intrinsic lifetime of the phosphors. The influence of lattice thermal vibration can be ignored. The mono-exponential (*n* = 1) and bi-exponential (*n* = 2) functions were used to fit their decay curves:^2^

2 $$I=\mathop{\sum }\limits_{i=1}^{n}{A}_{{\rm{i}}}\exp \left(-\frac{t}{{\tau }_{{\rm{i}}}}\right),\left(n=1,2,\cdots \right)$$

where *I* is the emission intensity at time *t*, *A*_i_ is fitting constants, and *τ*_i_ is the lifetime for different components. The decay curve of *x* = 1 is well fitted by both mono- and bi-exponential function, and the two fitting curves almost overlap. It is worth noting that only one type of Cr^3+^ luminescent center existed in *x* = 0, while its decay curve deviates from mono-exponential fitting and is well fitted by the bi-exponential function. This mismatch between the numbers of luminescent centers and fitting index is due to the additional nonradiative transitions^2^. One common explanation is energy migration between activator ions^44^, which is very likely to occur in this work. The results of DFT calculations, DR spectra, and XANES spectra demonstrate that Cr^3+^ and Cr^4+^ coexist in the synthesized phosphors, and the designed cosubstitution of [Zn^2+^–Zr^4+^] for [Y^3+^–Y^3+^] played a role as reductant to promote the valence reduction of Cr^4+^ to Cr^3+^ (from *x* = 0 to *x* = 1). The overlap between the PL spectrum of Cr^3+^ and the absorption spectrum of Cr^4+^ (inset of Fig. 2a) suggests the possibility of energy transfer in *x* = 0. According to the structural analysis, the YO_6_ octahedra are separated by GeO_4_ and CaO_8_ polyhedra. In *x* = 0, the distance between Y^3+^ and Ge^4+^ (3.58 Å) is much smaller than that between Y^3+^ and Y^3+^ (5.55 Å), which indicates that the distance between Cr^3+^ and Cr^4+^ is much smaller than that between Cr^3+^ and Cr^3+^. That is, the energy transfer can occur more easily between Cr^3+^ and Cr^4+^ rather than between Cr^3+^ and Cr^3+^. Thus, it is reasonable to ascribe the bi-exponential fitting for *x* = 0 phosphor to additional energy decay path caused by energy migration between Cr^3+^ and Cr^4+^. In *x* = 1, the concentration of Cr^4+^ is decreased, while that of Cr^3+^ is increased. This means the decreased Cr^3+^–Cr^4+^ energy transfer in *x* = 1. In addition, the distance between Cr^3+^ and Cr^3+^ is 5.44 Å, which exceeds the exchange interaction distance (<5 Å)^45^. Thus, the energy transfer process (from Cr^3+^ to Cr^4+^ and from Cr^3+^ to Cr^3+^) in *x* = 1 is limited to some extent. This could explain why the decay curve of *x* = 1 can be well-fitted by the mono-exponential function. The room temperature PL decay curves of Ca_3_Y_2-2*x*_(ZnZr)_*x*_Ge_3_O_12_:Cr (*x* = 0–1) phosphors were also recorded (Fig. S8a). Interestingly, the PL decay rate changes non-monotonically with *x*, but first exhibits a decreasing trend until *x* = 0.4 and then increase (Fig. S8b). To explain this, the decay curves were fitted by mono-exponential function. The resulted *R*-Squared (R^2^) and corresponding lifetime are shown in Fig. 2g. The R^2^ value first increases and then tends to be unchanged. The increased *R*^2^ means better mono-exponential fitting. On the one hand, the energy transfer from Cr^3+^ to Cr^4+^ decreases since Cr^4+^ concentration is lowered after the designed cosubstitution. On the other hand, the structure rigidity increases after cosubstitution, which can decrease the nonradiative transition from the lattice vibration at room temperature. These two aspects should result in the increased R^2^ value. However, the increased Cr^3+^ concentration as well as the shortened Cr^3+^–Cr^3+^ distance can increase the energy transfer between Cr^3+^ ions, as illustrated by Fig. 2h. Thus, the R^2^ value no longer increases at last. These results are consistent with the previous analysis about the reduction of Cr^4+^ to Cr^3+^ and improvement of structural rigidity through simple cosubstitution of [Zn^2+^–Zr^4+^] for [Y^3+^–Y^3+^]. As the energy transfer between Cr^3+^ and Cr^4+^ is more easily to occur than that between Cr^3+^ and Cr^3+^, the lifetime should increase with *x* as Cr^4+^ ions are transformed to Cr^3+^. However, this is not the case. The gradually introduced Zn^2+^ and Zr^4+^ ions coexist with Y^3+^ ions, which causes uneven crystal field around Cr^3+^, facilitating the breaking of d–d forbidden transition. This explains the shortened lifetime with *x* from 0 to 0.4. While the lifetime prolongation after *x* > 0.4 is ascribed to the gradually restored even field as well as the suppressed nonradiation transition by the increased structure rigidity. Figure 2i draws systematical models illustrating the effect of cosubstitution on chromium valence and crystal structure.

As analyzed above, cosubstitution of [Zn^2+^–Zr^4+^] for [Y^3+^–Y^3+^] in this system not only favor the formation of trivalent Cr^3+^, but also increases the lattice rigid. As a result, the NIR emission of Cr^3+^ is enhanced as expected. After cosubstitution, the internal quantum efficiency (IQE) is increased from 25% to 96%, and the external quantum efficiency (EQE) is increased from 6% to 20% (Fig. S9). The previously reported Ca_3_Y_2_Ge_3_O_12_:Cr^3+^ showed higher IQE (81%) and EQE (10%) than our Ca_3_Y_2_Ge_3_O_12_:Cr^3+^^46^, which is ascribed to the use of higher sintering temperature (1450 °C) and flux (LiF). It should be noted that using flux is also one of the methods to improve luminescence.

**Fig. 3:**
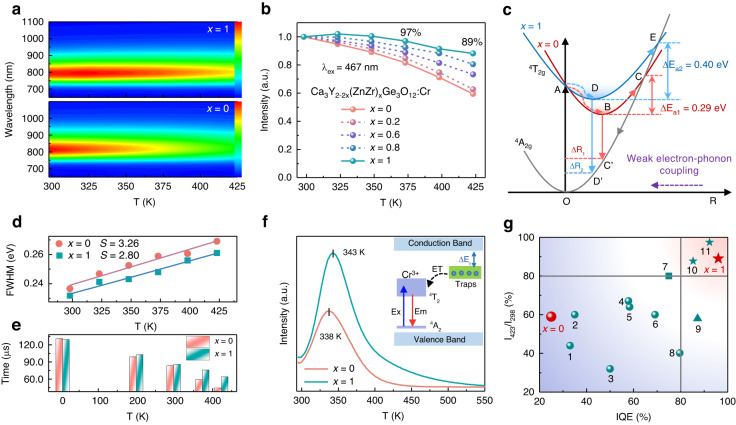
Temperature-dependent PL properties of Ca_3_Y_2-2x_(ZnZr)_*x*_Ge_3_O_12_**:Cr (*x*= 0–1).** **a** Temperature-dependent PL spectral profiles of *x* = 0 and *x* = 1. **b** Normalized emission intensity as a function of temperature of Ca_3_Y_2-2*x*_(ZnZr)_*x*_Ge_3_O_12_:Cr (*x* = 0–1). **c** Configuration coordinate curves illustrating the thermal quenching of Cr^3+^ luminescence. **d** Fitting results of FWHM as a function of *T* for *x* = 0 and *x* = 1. **e** Temperature-dependent PL decay times of *x* = 0 and *x* = 1. **f** TL curves of *x* = 0 and *x* = 1 measured in the temperature range of 295–550 K. The inset shows the charge transfer process from defects to emission centers. **g** Ratio of emission intensity under 423 and 298 K (I_423_/I_298_) and IQE for some Cr^3+^-doped garnet phosphors with emission peaking at 770–820 nm. The number 1–11 garnet phosphors are Ca_2_LaHf_2_Al_3_O_12_:Cr^3+^, La_3_Sc_2_Ga_3_O_12_:Cr^3+^, Gd_3_MgScGa_2_SiO_12_:Cr^3+^, Lu_2_CaMg_2_Ge_3_O_12_:Cr^3+^, Ca_2_LaZr_2_Ga_2.8_Al_0.2_O_12_:Cr^3+^, Ca_2_LuZr_2_Al_3_O_12_:Cr^3+^, Ca_2_YHf_2_Al_3_O_12_:Cr^3+^, Gd_3_Zn_0.8_Ga_3.4_Ge_0.8_O_12_:5%Cr^3+^, Na_3_In_2_Li_3_F_12_:Cr^3+^, Gd_3_In_2_Ga_3_O_12_:Cr^3+^, and Ca_3_Sc_2_Si_3_O_12_:Cr^3+^

As the high structural rigidity of the host means less soft phonon modes that cause nonradiative relaxation process, excellent PL thermal stability of the phosphors can be expected^47^. The thermal quenching resistance of Ca_3_Y_2-2*x*_(ZnZr)_*x*_Ge_3_O_12_:Cr (*x* = 0–1) is investigated (Fig. 3a and Fig. S10), which is greatly improved after the cosubstitution of [Zn^2+^–Zr^4+^] for [Y^3+^–Y^3+^]. As shown in Fig. 3b, *x* = 1 phosphor shows nearly zero-thermal quenching in the temperature range of 298–373 K. As the temperature elevates to 423 K, the emission intensity still keeps 89% of that at 298 K, which is much higher than that of 59% for *x* = 0. This confirms the positive effect of the designed cosubstitution on improving structure rigidity. The thermal quenching behavior can be expressed by the configuration coordinate diagram in Fig. 3c. Take *x* = 0 as an example, O→A and B→C’ represent the normal excitation and emission processes. The excited electrons are thermal activated by the elevated temperature to reach the intersection of the ^4^T_2g_ and ^4^A_2g_ curves (B→C) and then return to the ^4^A_2g_ ground state through nonradiative transition (C→O), leading to the so-called thermal quenching. The activation energy (*ΔE*_a_) indicates the difficulty of this thermal activated process, which can be determined by the Arrhenius formula^48^. The activation energy of *x* = 1 (0.40 eV) is higher than that of *x* = 0 (0.29 eV), in consistent with the better thermal stability of *x* = 1. The valence electrons of Cr^3+^ bared in outer layer have strong interactions with the lattice environment, which causes large lattice relaxation, display a horizontal displacement (*ΔR*) of the parabola in configuration coordinate diagram. It is clear that small displacement means large activation energy, which is related to rigid bond^49,50^. The rigid Cr–O bonds and second coordination shells cause the large activation energy for *x* = 1, increasing its thermal disturbance resistance. In addition, Huang’s theory points out that the activation energy is dependent on the strength of electron-phonon coupling, which can be reflected by the Huang–Rhys factor (*S*)^51,52^. The less spectral broadening with increasing temperature of *x* = 1 indicates its weaker electron-phonon coupling (Fig. S11). As shown in Fig. 2d, the factor *S* can be obtained by fitting the full-width at half maximum (FWHM) values of temperature-dependent PL spectra using the following equation:^51^

3 $${\rm{FWHM}}=2.36\sqrt{S}\hbar \omega \sqrt{\coth \left(\frac{\hbar \omega }{2{kT}}\right)}$$

where *ℏω* is the phonon energy, *k* is the Boltzmann constant, and *T* is the temperature. As a result, the phonon energy values for *x* = 0 and *x* = 1 are 0.048 and 0.052 eV, respectively. *S* values are obtained to be 3.26 for *x* = 0 and 2.80 for *x* = 1. The smaller *S* value for *x* = 1 implies that it has larger activation energy and higher thermal quenching temperature^52^. This also demonstrates the dependency of electron–phonon coupling and thermal quenching on structural rigidity. Structural rigidity could be further improved by adopting smaller host cations due to the shorter and more rigid bonds. However, a stronger crystal field would be introduced that generates shorter-wavelength emission. Hence, appropriately sized host cations are essential for high-performance long-wavelength NIR emission.

In order to further understand the different thermal quenching behaviors of *x* = 0 and *x* = 1, the temperature-dependent PL decay curves were measured (Fig. S12). Both of their PL decay rates accelerate with elevated temperature, which is attributed to the thermal-related nonradiative processes. It is observed from Fig. 3e that *x* = 1 shows slower decay rate than *x* = 0 with increasing temperature. In addition to the aforementioned influence of structural rigidity, the influence of thermally activated de-trapping of trapped electrons is also considered. XPS spectra of O 1 s show that oxygen vacancy defects exist in *x* = 0 and *x* = 1 (details are available in Supporting Information, Fig. S13), which form shallow traps below the conduction band. The trap depths are estimated to be 0.68 eV for *x* = 0 and 0.69 eV for *x* = 1 by the formula of *ΔE*_t_ = *T*_m_/500, where T_m_ represents the temperature of the peak center in thermoluminescence (TL) curves plotted in Fig. 3f^53,54^. At high temperature, the captured electrons in traps are released and charge transfer processes from defects to emission centers occur subsequently (see inset of Fig. 3f), which can compensate partial thermal loss of luminescence. The XPS results show that there are more oxygen vacancy defects in *x* = 1 (Fig. S13), which results in its higher TL intensity. The more traps in *x* = 1 can cause more emission compensation and observed better thermal quenching resistance. As shown in Fig. 3g, the excellent thermal stability and high IQE of *x* = 1 almost surpass all of the Cr^3+^-doped garnet phosphors within similar emission region (details are listed in Table S6)^24,25,30,55–62^. Overall, the EQE values of these Cr^3+^-doped garnet phosphors still need to be improved. Although the as-synthesized phosphors do not show advantage in EQE, this work achieved an improvement in photoluminescence quantum efficiency by the designed chemical unit cosubstitution. This demonstrates the feasibility of the idea about valence conversion and site reconstruction in improving luminescence for Cr^3+^-doped garnet phosphors.

### Chemical stability and information encryption

**Fig. 4:**
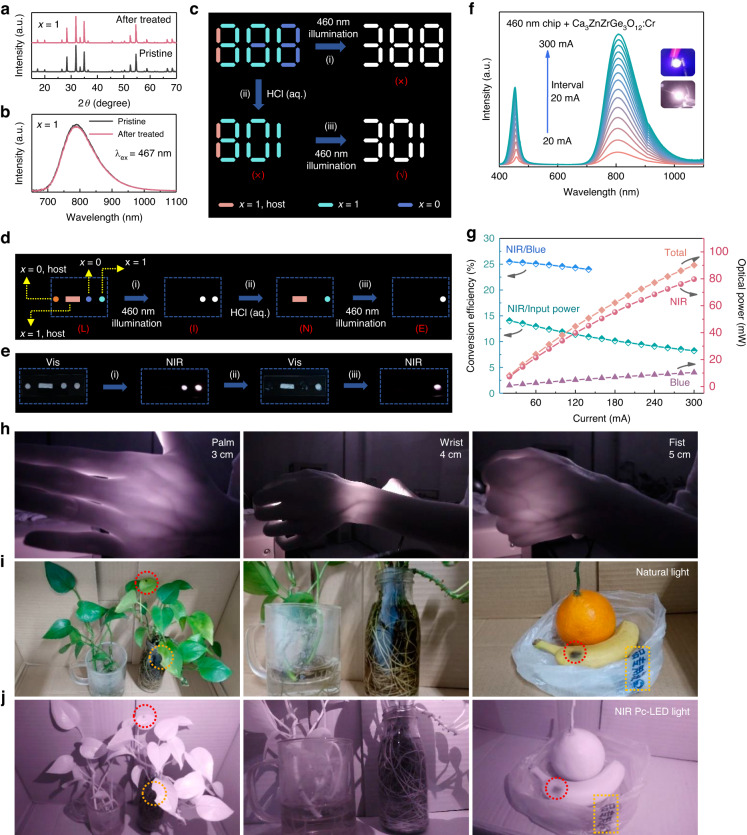
**Information encryption and pc-LED applications.** **a** XRD patterns and **b** PL spectra of *x* = 1 before (pristine) and after soaking in HCl (aq.) for 30 min. **c** Schematic diagram of the designed digital information encryption. The white “388” and “301” represent the NIR luminescent images. **d** Illustration of information encryption of “LINE” letters based on Morse code. The white dots represent the NIR luminescent images. **e** Practical demonstration of information encryption designed in Fig. 4d. The NIR luminescent images were captured by NIR camera. **f** Emission spectra and **g** NIR output power and photoelectric conversion efficiency under driving current in 20–300 mA of the as-fabricated pc-LED using *x* = 1 phosphor. The insets in Fig. 4f are photos of the as-fabricated pc-LED captured by visible and NIR camera, respectively. **h** Photos of the NIR pc-LED light penetrating palm, wrist, and fist captured by NIR camera. Photos of plants and fruits captured under **i** natural light by visible camera and **j** NIR pc-LED light by NIR camera

Previous works have focused on the thermal stability of phosphors, but have paid little attention to their chemical stability, which is also important to practical applications. Here, the influence of water, NaOH (aq.), and HCl (aq.) on *x* = 0 and *x* = 1 phosphors were investigated (details are described in Supporting Information, Fig. S14). Both *x* = 0 and *x* = 1 show good stability against water and strong base. However, *x* = 0 and *x* = 1 show extremely different chemical resistance to HCl (aq.). It was seen that *x* = 0 quickly decomposed in several seconds, while *x* = 1 showed better resistance (Fig. S15). As shown in Fig. 4a, b, *x* = 1 maintains the original phase and PL intensity after soaking in HCl (aq.) for 30 min. Even if it was placed in HCl (aq.) for 12 h, 64% PL intensity could still remain (Fig. S14d). The PL intensity loss is due to the partial destruction of phase by acid, as evidenced by Fig. S14e. This observation suggests more rigid covalent structure of *x* = 1 phosphor.

Inspired by the different chemical stability of phosphors in HCl (aq.), digital information encryption is designed. As shown in Fig. 4c, the real information is hidden in number “888” consist of by *x* = 0 phosphor, *x* = 1 phosphor, and *x* = 1 host. Under blue-light illumination, wrong information “388” is read out. After adding HCl (aq.) to the number “888”, the parts written by *x* = 0 phosphor disappear. Therefore, another interference information “801” is displayed. Only with the help of subsequent blue-light illumination, the real information “301” can be read. In addition, a coding method according to Morse code was developed to realize multiple information storage, as shown in Fig. 4d. The Morse code “dot” or “dash” are arranged as the designed coding pattern. After steps (i) – (iii), the information “LINE” can be decrypted. To demonstrate this, hosts *x* = 0, 1 and phosphors *x* = 0, 1 were mixed with proper amount of ethyl alcohol to form a slurry as inks, respectively. The Morse code “L” was written by the inks on a slide (Fig. 4e). After first blue-light illumination (step (i)), only two luminescent dots (Morse code “I”) were read. On step (ii), the two dots written by *x* = 0 host and phosphor disappeared in HCl (aq.). The rest one dash and one dot comprised the Morse code “N”. With blue-light illumination once again (step (iii)), only last one luminescent dot was seen. That is the Morse code “E”. This decryption process of information “LINE” can be completed within 1 min. Further, When HCl (aq.) was sprayed by simply heating, the initial Morse code “L” reappeared (Fig. S16). This reversible signal-switching process enables enhanced security for information encryption and achieves the purpose of “burning after reading”.

### LED package and applications

The optimal Ca_3_ZnZrGe_3_O_12_:Cr phosphor was coated on a blue chip (460 nm) to manufacture a NIR-emitting pc-LED. Figure 4f shows its driving current-dependent PL spectra. The emission peaks at around 460 and 800 nm is from the blue LED chip and the phosphor, respectively. It is seen from Fig. 4g that the output powers of total radiance, NIR, and blue light all increase with the increase of current in the range 20–300 mA. More details are listed in Table S7. The NIR output powers reach 34 and 80 mW under 100 and 300 mA, respectively. The conversion efficiency from blue LED to NIR light (*η*_NIR/blue_) decreases from 26% to 23% with current from 20 to 140 mA. The *η*_NIR/blue_ values under current beyond 140 mA are absent since the output power of blue LED is out of maximum range of the instrument. The conversion efficiency from input electrical power to NIR light (*η*_NIR/input_) drops from 14% to 7% with current from 20 to 300 mA, which is much lower than *η*_NIR/blue_. This should be ascribed to the unsatisfied photoelectric conversion efficiency from input electrical power to blue light (Table S8). The as-fabricated pc-LED still show comparable photoelectric performance to the reported (Table S6). Once the blue chips become efficient, the *η*_NIR/input_ and NIR output power can be further enhanced. As show in Fig. S17a, the emission intensity of the pc-LED only slightly decreases after it being placed in air for 6 months. In addition, after continuous measurement at 100 mA for 12 h, the NIR emission intensity of the pc-LED remains almost unchanged (Fig. S17b). These two results indicate the remarkable stability of the fabricated pc-LED. Figure 4h shows the images of the pc-LED light penetrating palm, wrist, and fist captured by NIR camera. Blood vessels still can be clearly observed when the penetrate depth is 5 cm, which indicates that the as-fabricated NIR-emitting pc-LED is promising for nondestructive examination of deep bio-tissues. The application ability of this pc-LED in night vision is also investigated. Figure 4i shows the photographs of plants and fruits taken by visible camera under natural light. Figure 4j shows the corresponding clear photographs taken by NIR camera under the NIR pc-LED light. Even the small black dot and gap on the leaves, rhizomes of the plants, black spot on the banana peel, and the blue Chinese characters on the plastic bag can be distinguished. These results demonstrate that our NIR pc-LED has great application potential in night vision techniques.

## Discussion

In summary, NIR-emitting Ca_3_Y_2-2*x*_(ZnZr)_*x*_Ge_3_O_12_:Cr garnet solid-solution system was established by the designed chemical unit cosubstitution of [Zn^2+^–Zr^4+^] for [Y^3+^–Y^3+^], which significantly improved the IQE from 25% to 96% and thermal stability (*I*_423 K_/*I*_298 K_) from 59% to 89%, with only slight emission shift from 812 to 795 nm. The overlarge size-mismatch between Cr^3+^ and Y^3+^ contributed to the coexistence of Cr^3+^ and Cr^4+^ in Ca_3_Y_2_Ge_3_O_12_:Cr, which quenched the luminescence of Cr^3+^. As suggested by DFT calculations, the introduction of smaller [Zn^2+^–Zr^4+^] units to Y sites could reconstruct the octahedral sites for Cr^3+^ ions to promote their formation in Ca_3_ZnZrGe_3_O_12_:Cr, which was responsible for the enhanced luminescence. Moreover, the reconstructed rigid structure also contributed to the high photoluminescence quantum efficiency and thermal stability. Benifing from the weakened electron-phonon coupling effect and the luminescence compensation by traps, Ca_3_ZnZrGe_3_O_12_:Cr exhibited nearly zero-thermal quenching at 373 K and 11% thermal quenching loss at 423 K. In addition, Both Ca_3_Y_2_Ge_3_O_12_:Cr and Ca_3_ZnZrGe_3_O_12_:Cr showed outstanding water and base resistance, but showed different acid resistance. Information storage was accordingly designed inspired by this difference. Finally, the pc-LED fabricated by Ca_3_ZnZrGe_3_O_12_:Cr demonstrated great potential in bio-tissue imaging and night-vision technologies. This work provides a new perspective of luminescence optimization by chemical unit cosubstitution and could motivate further exploration of high-performance Cr^3+^-doped garnet phosphors and other types of NIR-emitting phosphor materials.

## Materials and methods

### Materials synthesis

The designed composition of Ca_3_Y_2-2*x*_(ZnZr)_*x*_Ge_3_O_12_:Cr (*x* = 0–1) phosphors is Ca_3_Y_1.99-2*x*_(ZnZr)_*x*_Ge_3_O_12_:0.01Cr, which were synthesized by a high-temperature solid-state reaction method. Calcium carbonate (CaCO_3_, 99.99%), yttrium(III) oxide (Y_2_O_3_, 99.99%), zirconium dioxide (ZrO_2_, 99.99%), germanium dioxide (GeO_2_, 99.99%), and chromium sesquioxide (Cr_2_O_3_, 99.95%) were obtained from Aladdin Reagent Co., Ltd. Zinc oxide (ZnO, A. R.) was acquired from Beijing Chemical Works. Stoichiometrical raw materials were weighed and thoroughly ground in agate mortars for 20 min. Then the mixtures were put into alumina crucibles and sintered at 1673 K for 6 h in a box furnace. The resulting products were slowly cooled down to room temperature and ground again for further characterization.

### LED fabrication

The optimal Ca_3_ZnZrGe_3_O_12_:Cr NIR-emitting phosphor was firstly mixed with epoxy resins A and B (mass ratio of 1:1) and then coated on the 460 nm blue chips. The mixtures were cured at 100 °C for 1 h to form the final LED devices.

### Characterization

The XRD patterns of the phosphors were measued on a Bruker D8 ADVANCE powder diffractometer using Cu Kα radiation (*λ* = 1.54 Å) in the 2*θ* range of 10°−120°. XRD Rietveld refinements were conducted using the GSAS program to reveal the crystal structure and phase purity. The morphology and elemental mapping were obtained by the field-emission scanning electron microscope (FE-SEM, S-4800, Hitachi) equipped with an Energy Dispersive Spectrometer (EDS). Raman spectra were measured by a Raman spectrometer (Model T64000, Horiba JobinYvon, France) using a 512 nm laser. The diffuse reflectance (DR) spectra were recorded on a UV−vis−NIR spectrophotometer (UV-3600 plus, Shimadzu, Japan). The photoluminescence excitation (PLE) and photoluminescence (PL) spectra were measured by the fluorescence spectrometer (Edinburgh Instruments FLSP-920) with a 450 W xenon lamp as excitation source. The light detector R5509-72 photomultiplier (PMT) was used. The PL decay curves were measured by an Edinburgh Instruments FLSP-920 fluorescence spectrometer with a μF2 lamp as excitation source. The temperature-dependent PL spectra and decay curves were also recorded by Edinburgh Instruments FLSP-920 fluorescence spectrometers equipped with a temperature controller. The quantum efficiency measurement was carried out on the measurement system (C9920-02, Hamamatsu photonics K. K., Japan) equipped with a 150 W Xe lamp. The X-ray absorption experiments were carried out at the XAS station (BL14W1) of the Shanghai Synchrotron Radiation Facility. The X-ray photoelectron spectroscopy (XPS) spectra were measured using the Thermo SCIENTIFIC ESCALAB 250Xi with an Al Kα source. The thermoluminescence (TL) spectra were obtained from an LTTL-3DS measurement with a heating rate of 2 K/s. Before TL measurement, each sample was pre-irradiated with a UV lamp (254 nm) for 5 min. The emission spectrum of the as-fabricated pc-LED was measured on the HAAS 2000 photoelectric measuring system from EVERFINE.

### Theoretical calculations

Theoretical calculations were performed using density functional theory (DFT) implemented in Vienna Ab-initio Simulation Package (VASP)^63^. The generalized gradient approximation (GGA) with the PBE functional was applied. The cutoff energy and *k*-point mesh were set to 450 eV and the 3 × 3 × 3 Monkhorst-Pack grid, respectively. The convergence criterion for the electronic energy was 10^−5^ eV and the structures were relaxed until the Hellmann–Feynman forces were smaller than 0.02 eVÅ^−1^.

The Debye temperature *Θ*_D_ was calculated based on the harmonic Debye model, relying on the bulk modulus and Poisson ratio:^64^

4 $${\varTheta }_{{\rm{D}}}=\frac{\hbar }{{k}_{{\rm{B}}}}{\left[6{\pi }^{2}{V}^{1/2}N\right]}^{1/3}\sqrt{\frac{{B}_{{\rm{H}}}}{M}}f\left(v\right)$$

5 $$f\left(v\right)={\left\{{\left[{2\left(\frac{2}{3}\cdot \frac{1+v}{1-2v}\right)}^{3/2}+{\left(\frac{1}{3}\cdot \frac{1+v}{1-v}\right)}^{3/2}\right]}^{-1}\right\}}^{1/3}$$

where *k*_B_ and ℏ are the Boltzmann constant and Planck constant, respectively. *B*_H_ represents the adiabatic bulk modulus of the crystal, *M* is the molecular mass of the unit cell, *N* means the number of atoms per unit cell, *V* is the unit cell volume, and *v* is the Poisson ratio.

The formation energy (*E*_f_) of the Cr^3+^ and Cr^4+^ doped Ca_3_Y_2_Ge_3_O_12_ and Ca_3_ZnZrGe_3_O_12_ can be calculated by^63^:

6 $${E}_{{\rm{f}}}=E\left({\rm{doped}}\right)-E\left({\rm{perfect}}\right)-\sum {n}_{{\rm{i}}}{{\rm{\mu }}}_{{\rm{i}}}$$

where *E*(doped) and *E*(perfect) are the total energy of the doped and undoped crystal, respectively. The *n*_i_ and *μ*_i_ represent the chemical potential and the number of the added (*n*_i_ > 0) or removed (*n*_i_ < 0) *i*-type atoms, respectively.

### Supplementary information

Supplementary Information
