# Supplementary material for: Valence conversion and site reconstruction in near-infrared-emitting chromium-activated garnet for simultaneous enhancement of quantum efficiency and thermal stability
Source: Light Sci Appl. 2023 Oct 7;12:248. doi: 10.1038/s41377-023-01283-3 (PMC10560275; doi:10.1038/s41377-023-01283-3)
Supplement: Supplementary file 1 — Supplementary Information [file 41377_2023_1283_MOESM1_ESM.docx]

Supplementary Information for

Valence conversion and site reconstruction in near-infrared-emitting chromium-activated garnet for simultaneous enhancement of quantum efficiency and thermal stability

Dongjie Liu,^1^ Guogang Li,^2,3,^* Peipei Dang,^1^ Qianqian Zhang,^1,4^ Yi Wei,^2^ Lei Qiu,^2^ Hongzhou Lian,^1^ Mengmeng Shang,^5^ and Jun Lin^1,4,^*

^1^ State Key Laboratory of Rare Earth Resource Utilization, Changchun Institute of Applied Chemistry, Chinese Academy of Sciences, Changchun 130022, China. E-mail: jlin@ciac.ac.cn

^2^ Faculty of Materials Science and Chemistry, China University of Geosciences, Wuhan 430074, China. E-mail: ggli@cug.edu.cn

^3^ Zhejiang Institute, China University of Geosciences, Hangzhou, 311305, China

^4^ University of Science and Technology of China, Hefei 230026, China

^5^ School of Material Science and Engineering, Shandong University, Jinan 266071, China

**Table S1.** Ionic radii for cation ions under corresponding coordination numbers.

| Ion | Coordination number (CN) | Ionic radius (Å) |
| --- | --- | --- |
| Y^3+^ | 6 | 0.9 |
| Zn^2+^ | 6 | 0.74 |
| Zr^4+^ | 6 | 0.72 |
| Cr^3+^ | 6 | 0.615 |
| Ge^4+^ | 4 | 0.39 |
| Cr^4+^ | 4 | 0.41 |

**Fig. S1** XRD Rietveld refinements of Ca_3_Y_2-2_*_x_*(ZnZr)*_x_*Ge_3_O_12_:Cr (*x* = 0–0.8).

**Table S2.** Main parameters of processing and refinement of Ca_3_Y_2-2_*_x_*(ZnZr)*_x_*Ge_3_O_12_:Cr (*x* = 0–1).

| Sample | *x* = 0 | *x* = 0.2 | *x* = 0.4 | *x* = 0.8 | *x* = 1 |
| --- | --- | --- | --- | --- | --- |
| Space group | *Ia*-3*d* | *Ia*-3*d* | *Ia*-3*d* | *Ia*-3*d* | *Ia*-3*d* |
| *a = b = c*, Å | 12.80778 (4) | 12.77179 (7) | 12.70901 (21) | 12.60694 (9) | 12.57619 (4) |
| *V*, Å^3^ | 2100.979 (11) | 2083.318 (19) | 2052.75 (6) | 2003.684 (25) | 1989.055 (12) |
| *α* = *β* = *γ*, º | 90 | 90 | 90 | 90 | 90 |
| *Z* | 8 | 8 | 8 | 8 | 8 |
| *2θ*-interval, º | 10-120 | 10-120 | 10-120 | 10-120 | 10-120 |
| *R*_wp_, % | 9.36 | 7.78 | 8.96 | 9.93 | 9.41 |
| *R*_p_, % | 6.56 | 5.57 | 6.64 | 7.68 | 6.83 |
| *χ^2^* | 7.48 | 3.63 | 5.82 | 6.45 | 5.92 |

**Table S3.** Main bond lengths (Å) of Ca_3_Y_2-2_*_x_*(ZnZr)*_x_*Ge_3_O_12_:Cr (*x* = 0–1).

| *x* = 0 | | | | | |
| --- | --- | --- | --- | --- | --- |
| Ca–O | 2.53153 (1) | Y–O | 2.23662 (1) | Ge–O | 1.78338 (0) |
| Ca–O | 2.48084 (1) | Y–O | 2.23662 (1) | Ge–O | 1.78338 (0) |
| Ca–O | 2.53153 (1) | Y–O | 2.23662 (1) | Ge–O | 1.78338 (0) |
| Ca–O | 2.48084 (1) | Y–O | 2.23662 (1) | Ge–O | 1.78338 (0) |
| Ca–O | 2.48084 (1) | Y–O | 2.23662 (1) |  |  |
| Ca–O | 2.53153 (1) | Y–O |  |  |  |
| Ca–O | 2.53153 (1) |  |  |  |  |
| Ca–O | 2.48084 (1) |  |  |  |  |
| Average | 2.50619 | Average | 2.23662 | Average | 1.78338 |
| *x* = 0.2 | | | | | |
| Ca–O | 2.52324 (1) | Y/Zn/Zr–O | 2.24927 (1) | Ge–O | 1.79116 (1) |
| Ca–O | 2.41464 (1) | Y/Zn/Zr–O | 2.24927 (1) | Ge–O | 1.79116 (1) |
| Ca–O | 2.52324 (1) | Y/Zn/Zr–O | 2.24927 (1) | Ge–O | 1.79116 (1) |
| Ca–O | 2.41464 (1) | Y/Zn/Zr–O | 2.24927 (1) | Ge–O | 1.79116 (1) |
| Ca–O | 2.41464 (1) | Y/Zn/Zr–O | 2.24927 (1) |  |  |
| Ca–O | 2.52324 (1) | Y/Zn/Zr–O | 2.24927 (1) |  |  |
| Ca–O | 2.52324 (1) |  |  |  |  |
| Ca–O | 2.41464 (1) |  |  |  |  |
| Average | 2.46894 | Average | 2.24927 | Average | 1.79116 |
| *x* = 0.4 | | | | | |
| Ca–O | 2.51447 (4) | Y/Zn/Zr–O | 2.21635 (3) | Ge–O | 1.78125 (2) |
| Ca–O | 2.43434 (3) | Y/Zn/Zr–O | 2.21635 (3) | Ge–O | 1.78125 (2) |
| Ca–O | 2.51447 (4) | Y/Zn/Zr–O | 2.21635 (3) | Ge–O | 1.78125 (2) |
| Ca–O | 2.43434 (3) | Y/Zn/Zr–O | 2.21635 (3) | Ge–O | 1.78125 (2) |
| Ca–O | 2.43434 (3) | Y/Zn/Zr–O | 2.21635 (3) |  |  |
| Ca–O | 2.51447 (4) | Y/Zn/Zr–O | 2.21635 (3) |  |  |
| Ca–O | 2.51447 (4) |  |  |  |  |
| Ca–O | 2.43434 (3) |  |  |  |  |
| Average | 2.47441 | Average | 2.21635 | Average | 1.78125 |
| *x* = 0.8 | | | | | |
| Ca–O | 2.54713 (2) | Y/Zn/Zr–O | 2.17340 (1) | Ge–O | 1.74669 (1) |
| Ca–O | 2.40781 (1) | Y/Zn/Zr–O | 2.17340 (1) | Ge–O | 1.74669 (1) |
| Ca–O | 2.54713 (2) | Y/Zn/Zr–O | 2.17340 (1) | Ge–O | 1.74669 (1) |
| Ca–O | 2.40781 (1) | Y/Zn/Zr–O | 2.17340 (1) | Ge–O | 1.74669 (1) |
| Ca–O | 2.40781 (1) | Y/Zn/Zr–O | 2.17340 (1) |  |  |
| Ca–O | 2.54713 (2) | Y/Zn/Zr–O | 2.17340 (1) |  |  |
| Ca–O | 2.54713 (2) |  |  |  |  |
| Ca–O | 2.40781 (1) |  |  |  |  |
| Average | 2.47747 | Average | 2.17340 | Average | 1.74669 |
| *x* = 1 | | | | | |
| Ca–O | 2.53671 (1) | Zn/Zr–O | 2.14815 (1) | Ge–O | 1.76352 (0) |
| Ca–O | 2.39698 (1) | Zn/Zr–O | 2.14815 (1) | Ge–O | 1.76352 (0) |
| Ca–O | 2.53671 (1) | Zn/Zr–O | 2.14815 (1) | Ge–O | 1.76352 (0) |
| Ca–O | 2.39698 (1) | Zn/Zr–O | 2.14815 (1) | Ge–O | 1.76352 (0) |
| Ca–O | 2.39698 (1) | Zn/Zr–O | 2.14815 (1) |  |  |
| Ca–O | 2.53671 (1) | Zn/Zr–O | 2.14815 (1) |  |  |
| Ca–O | 2.53671 (1) |  |  |  |  |
| Ca–O | 2.39698 (1) |  |  |  |  |
| Average | 2.46685 | Average | 2.14815 | Average | 1.76352 |

**Fig. S2** SEM images of **a** *x* = 0 and **b–d** *x* = 1.

**Fig. S3** **a** PL spectra and **b** normalized PL spectra of Ca_3_Y_2-_*_y_*Ge_3_O_12_:*y*Cr.

With the increase of Cr concentration, the emission intensity shows a trend of first rising and then falling due to the concentration quenching, reaching a maximum at *y* = 0.01 (Fig. S3a). The various Cr concentration show little influence on emission wavelength. Only very slight emission red-shift is observed (Fig. S3b). Although doping smaller Cr into the host can increase the crystal field splitting parameter (*D*_q_), the larger electronegativity of Cr can cause the increased Racah parameter *B*. As a result, the crystal field (*D*_q_/*B*) remains almost unchanged.

**Fig. S4** **a** Normalized PLE spectra, **b** relative PL intensity, and **c** normalized PL spectra of Ca_3_Y_2-2_*_x_*(ZnZr)*_x_*Ge_3_O_12_:Cr (*x* = 0–1).

**Fig. S5** Calculated crystal field strength parameter *D*_q_, Racah parameter *B*, and *D*_q_/*B* for Ca_3_Y_2-2x_(ZnZr)*_x_*Ge_3_O_12_:Cr (*x* = 0–1).

**Fig. S6** Tanabe–Sugano diagram for d^3^ electronic configuration of Cr^3+^ in an octahedral crystal field.

**Table S4.** Transition energy values and calculated *D*_q_, *B*, and *D*_q_/*B*.

| Phosphor | ^4^A_2g_-^4^T_1g_ (cm^-1^) | ^4^A_2g_-^4^T_2g_ (cm^-1^) | *D*_q_ (cm^-1^) | *B* (cm^-1^) | *D*_q_/*B* |
| --- | --- | --- | --- | --- | --- |
| *x* = 0 | 21008 | 14646 | 1464.6 | 656.8 | 2.23 |
| *x* = 0.2 | 21060 | 14763 | 1476.3 | 644.7 | 2.29 |
| *x* = 0.4 | 21192 | 14913 | 1491.3 | 640 | 2.33 |
| *x* = 0.6 | 21253 | 14979 | 1497.9 | 637 | 2.35 |
| *x* = 0.8 | 21277 | 15009 | 1500.9 | 636 | 2.36 |
| *x* = 1 | 21336 | 15102 | 1510.2 | 629.3 | 2.40 |

To understand the blue shift of *d*–*d* transition for Cr^3+^, the crystal field strength *D*_q_ and the Racah parameters *B* can be obtained using following equations:^1^

$$D_{q}=\frac{E\left( {}^{4}{A_{2g}}-{}^{4}{T_{2g}} \right)}{10}$$

(S1)

$$\frac{D_{q}}{B}=\frac{15\left( \frac{\Delta E_{4T}}{D_{q}}-8 \right)}{\left( \frac{\Delta E_{4T}}{D_{q}} \right)^{2}-10\left( \frac{\Delta E_{4T}}{D_{q}} \right)}$$

(S2)

$$\Delta E_{4T}=E\left( {}^{4}{T_{1g}} \right)-E\left( {}^{4}{T_{2g}} \right)$$

(S3)

As a result, *D*_q_ and *B* increases and decreases with increasing *x*, respectively (Table S4). The contraction of Cr^3+^–O^2-^ bonds is responsible for the increased *D*_q_ based on the following formula:^2^

$$D_{q}=\frac{1}{6}Ze^{2}\frac{r^{4}}{R^{5}}$$

(S4)

where *Z* is the anion valence, *e* is the electron charge, *r* is the radius of the *d* wave function, and *R* is the bond length. The Racah parameter *B* is related to the covalency, which increases with reducing covalency between central ions and ligands.^3^ The contraction of Cr^3+^–O^2-^ bonds expands the overlap of the electron clouds between Cr^3+^ and O^2-^, leading to the stronger Cr^3+^–O^2-^ covalent bonds and reducing the Racah parameter *B*. Thus, the crystal field (*D*_q_/*B*) is strengthened. The above analysis explains the reasonable blue shift in PL spectra, as interpreted by Tanabe–Sugano diagram in Figure S6.

**Fig. S7** **a** PL and **b** normalized PLE spectra of *x* = 0 phosphor measured under 7 K.

**Table S5.** The vibration modes of activated Raman peaks in *x* = 1 phosphor.

| Band | Raman modes (cm^−1^) | Symmetry modes | Assignments |
| --- | --- | --- | --- |
| 1 | 175 | T_2g_ | Ca^2+^_translation_ |
| 2 | 258 | E_g_ | Ca^2+^_translation_ |
| 3 | 295 | E_g_ + T_2g_ | [GeO_4_]_translation_ + [GeO_4_]_rotatory_ |
| 4 | 335 | T_2g_ | [GeO_4_]_rotatory_ |
| 5 | 480 | E_g_ | [GeO_4_]_bending_ |
| 6 | 550 | E_g_ | [GeO_4_]_bending_ |
| 7 | 686 | T_2g_ | [GeO_4_]_bending_ |
| 8 | 724 | T_2g_ | [GeO_4_]_stretching_ |
| 9 | 780 | T_2g_ | [GeO_4_]_stretching_ |
| 10 | 801 | A_1g_ | [GeO_4_]_stretching_ |
| 11 | 820 | T_2g_ | [GeO_4_]_stretching_ |
| 12 | 850 | A_1g_ | [GeO_4_]_stretching_ |

Based on the group theory analysis, a cubic garnet structure with space group *Ia*-3*d* presents 25 Raman active modes and can be summarized as follows:^4,5^

*Γ*_Raman_ = 3*A*_1g_ + 8*E*_g_ + 14*T*_2g_

(S5)

where A_1g_, E_g_ and T_2g_ denote the internal, translational, and rotational modes, respectively. These modes are originated from different vibrations of CaO_8_ and GeO_4_, as listed in Table S5. However, not all 25 Raman modes could be detected, which may be due to the peak overlapping or low instrument resolution.

**Fig. S8 a** PL decay curves of Ca_3_Y_2-2_*_x_*(ZnZr)*_x_*Ge_3_O_12_:Cr (*x* = 0–1) phosphors. **b** Converted contour map of PL decay curves. These decay curves were excited under 470 nm, monitoring at corresponding emission wavelengths, which are 812, 809, 807, 804, 800, and 795 nm for *x* = 0, 0.2, 0.4, 0.6, 0.8, and 1, respectively.

**Fig. S9** QE measuring spectra of **a** *x* = 0 and **b** *x* = 1 phosphors. Comparison between QE measuring spectra and PL spectra for **c** *x* = 0 and **d** *x* = 1 phosphors.

The luminescent spectra for QE measurement (S_1_ part) were only recorded till 950 nm due to the range limitation of the instrument. The missing S_2_ part (950–1100) should be taken into consideration for the actual IQE. Therefore, the actual IQE can be calculated via the following equation:^6^

$$IQE={IQE}_{m}\times\left( S_{1}+S_{2} \right)/{S_{1}}$$

(S6)

where IQE and IQE_m_ are the actual IQE and measured IQE. S_1_ (650–950 nm) and S_2_ (950–1100 nm) are integrated intensities, which are obtained from PL spectra. The actual IQEs of *x* = 0 and *x* = 1 phosphors are determined to be 25% and 96%, respectively. The absorption efficiencies (Abs) of *x* = 0 and *x* = 1 are 24% and 21%, respectively. The external quantum efficiencies (EQE) are calculated to be 6% for *x* = 0 and 20% for *x* = 1, according to the following equation:^7^

$$EQE =IQE\times Abs$$

(S7)

**Fig. S10** Temperature-dependent PL spectra of Ca_3_Y_2-2_*_x_*(ZnZr)*_x_*Ge_3_O_12_:Cr^3+^ (*x* = 0–1) in the temperature range of 298–423 K.

**Fig. S11** Normalized temperature-dependent PL spectra of *x* = 0 and *x* = 1 phosphors.

**Fig. S12** Temperature-dependent PL decay curves of **a** *x* = 0 and **b** *x* = 1 in the temperature range of 7–423 K.

**Fig. S13** XPS spectra of O 1s for *x* = 0 and *x* = 1 phosphors.

The O 1s XPS peak of *x* = 0 and *x* = 1 phosphors could be fitted by four Gaussian peaks, centered at about 530.1, 531.0, 532.0, and 533.0 eV. The high binding energy components located at 532.0 (peak 2) and 533.0 (peak 1) eV are usually attributed to the presence of loosely bound oxygen such as adsorbed O_2_, H_2_O, and CO_2_.^8^ Two types of O^2-^ ions can be distinguished on the low binding energy side, O_I_ (peak 3) and O_II_ (peak 4). The O_II_^2-^ ions have neighboring atoms with full six nearest O^2-^ ions, while the O_I_^2-^ ions are in oxygen-deficient regions.^9^ Therefore, the relative intensity of O_I_ / O_II_ are related to the concentration of oxygen vacancies.^9^ The *x* = 1 phosphor shows higher relative intensity of O_I_ / O_II_, indicating more oxygen vacancies were generated in it.

**Table S6.** Emission intensity ratio of 423 K and 298 K (I_423_/I_298_) and quantum efficiency (IQE and EQE) for some Cr^3+^-doped garnet phosphors with emission wavelength in 770–820 nm, as well as NIR output power and conversion efficiency of the corresponding fabricated pc-LEDs.

| No. | Phosphor | λ_em_ (nm) | IQE  (%) |  | EQE  (%) | I_423_/I_298_  (%) | NIR output power | Conversion efficiency (%) | Ref |
| --- | --- | --- | --- | --- | --- | --- | --- | --- | --- |
| 1 | Ca_2_LaHf_2_Al_3_O_12_:Cr^3+^ | 780 | 33 |  | / | 44 | 12 mW @ 100 mA | 10 | ^10^ |
| 2 | La_3_Sc_2_Ga_3_O_12_:Cr^3+^ | 818 | 35 |  | / | 60 | / | / | ^11^ |
| 3 | Gd_3_MgScGa_2_SiO_12_:Cr^3+^ | 820 | 50 |  | / | 32 | 20 mW @ 100 mA | 7.7 | ^12^ |
| 4 | Lu_2_CaMg_2_Ge_3_O_12_:Cr^3+^ | 795 | 57.8 |  | 26.8 | 67.1 | 36 mW @ 100 mA | 13 | ^13^ |
| 5 | Ca_2_LaZr_2_Ga_2.8_Al_0.2_O_12_:Cr^3+^ | 820 | 58.3 |  | 23.5 | 64 | 32.2 mW @ 100 mA | 11.6 | ^14^ |
| 6 | Ca_2_LuZr_2_Al_3_O_12_:Cr^3+^ | 780 | 69.1 |  | / | 60 | / | / | ^15^ |
| 7 | Ca_2_YHf_2_Al_3_O_12_:Cr^3+^ | 775 | 75 |  | / | 80 | 2.71 mW @ 20 mA | 5.2 | ^16^ |
| 8 | Gd_3_Zn_0.8_Ga_3.4_Ge_0.8_O_12_:5%Cr^3+^ | 800 | 79.6 |  | 31.2 | 40.2 | / | / | ^17^ |
| 9 | Na_3_In_2_Li_3_F_12_:Cr^3+^ | 778 | 87.2 |  | 20.1 | 58 | 47.92 mW @ 100 mA | 17.6 | ^18^ |
| 10 | Gd_3_In_2_Ga_3_O_12_:Cr^3+^ | 780 | 85.3 |  | 42 | 87.7 | 33.7 mW @ 100 mA | 12.0 | ^19^ |
| 11 | Ca_3_Sc_2_Si_3_O_12_:Cr^3+^ | 770 | 92.3 |  | 25.5 | 97.4 | 23.7 mW @ 100 mA | 8.5 | ^20^ |
| 12 | Ca_3_Y_2_Ge_3_O_12_:Cr^3+^ | 800 | 81 |  | 10 | / | / |  | ^21^ |
| *x* = 0 | Ca_3_Y_2_Ge_3_O_12_:Cr^3+^ | 812 | 25 |  | 6 | 56 | / | / | This work |
| *x* = 1 | Ca_3_ZnZrGe_3_O_12_:Cr^3+^ | 795 | 96 |  | 20 | 89 | 34 mW @ 100 mA | 12 | This work |
|  |  |  |  |  |  |  | 7.3 mW @ 20 mA | 14 |  |

**Fig. S14** XRD patterns of **a** *x* = 0 and **b** *x* = 1 phosphors before (pristine) and after soaking in H_2_O and NaOH (aq.) for 24 h. Relative PL intensity of **c** *x* = 0 and **d** *x* = 1 phosphors before (pristine) and after soaking in H_2_O and NaOH (aq. 1 M) for 24 h, and after soaking in HCl (aq.) for 12 h. **e** XRD patterns of *x* = 1 phosphor before (pristine) and after soaking in HCl (aq.) for 12 h.

Here, the chemical resistance of *x* = 0 and *x* = 1 phosphors to water, NaOH (aq., 1 M), and HCl (aq., concentrated hydrochloric acid diluted with equal volume of water) were evaluated. When *x* = 0 and *x* = 1 phosphors were soaked in water and NaOH (aq.) for 24 h, their XRD phases remained unchanged compared the pristine phases (Figures S14a–b). Moreover, the PL spectra *x* = 0 and *x* = 1 phosphors almost maintained the original intensities (Figures S14c–d). However, *x* = 0 phosphor was extremely instable in strong acid, which quickly decomposed in HCl (aq.) only in several seconds, while *x* = 1 showed better resistance to HCl (aq.) (Figure S15). When *x* = 1 phosphor was placed in HCl (aq.) for 12 h, 64% PL intensity could still remain (Figure S14d). The intensity loss is ascribed to the phase destruction by acid, as evidenced by Figure S14e.

**Fig. S15** Photos of **a** *x* = 0 and **b** *x* = 1 phosphors before and after soaking in HCl (aq.). It is noted that the *x*= 0 phosphor is quickly decomposed by HCl (aq.).

**Fig. S16 a** Photos of changes in designed coding pattern by adding HCl (aq.) and following heating. **b** comparison of XRD patterns of the reappeared parts (after heating) to corresponding pristine in initial Morse code “L”.

It should be illustrated that the compositions of the reappeared two dots after heating are different from the two in the initial Morse code “L”, as the XRD pattern of the reappeared parts is totally different from pristine. The pristine has been decomposed by HCl (aq.). Therefore, the reappeared coding pattern cannot repeat the decryption process again, which interferes with the secondary decryption process and realizes the purpose of “burning after reading”.

**Table S7.** Detailed performances of the fabricated NIR-emitting pc-LED.

| Current (mA) | Voltage  (V) | luminous flux (lm) | luminous efficacy (lm W^−1^) | Blue output power  (mW) | NIR output power (mW) | NIR photoelectric conversion efficiency (%) |
| --- | --- | --- | --- | --- | --- | --- |
| 20 | 2.63 | 0.05 | 1.03 | 0.85 | 7.34 | 14.0 |
| 40 | 2.70 | 0.10 | 0.96 | 1.72 | 14.57 | 13.5 |
| 60 | 2.76 | 0.15 | 0.9 | 2.57 | 21.43 | 13.0 |
| 80 | 2.82 | 0.19 | 0.84 | 3.38 | 27.89 | 12.4 |
| 100 | 2.86 | 0.23 | 0.79 | 4.15 | 33.98 | 11.9 |
| 120 | 2.91 | 0.26 | 0.75 | 4.90 | 39.79 | 11.4 |
| 140 | 2.95 | 0.29 | 0.71 | 5.61 | 45.12 | 10.9 |
| 160 | 2.99 | 0.33 | 0.68 | 6.30 | 50.51 | 10.6 |
| 180 | 3.03 | 0.35 | 0.65 | 6.95 | 55.25 | 10.1 |
| 200 | 3.06 | 0.38 | 0.62 | 7.57 | 60.07 | 9.8 |
| 220 | 3.10 | 0.41 | 0.60 | 8.18 | 64.30 | 9.4 |
| 240 | 3.13 | 0.43 | 0.58 | 8.76 | 68.40 | 9.1 |
| 260 | 3.16 | 0.46 | 0.56 | 9.32 | 72.42 | 8.8 |
| 280 | 3.19 | 0.48 | 0.54 | 9.89 | 76.10 | 8.5 |
| 300 | 3.22 | 0.51 | 0.52 | 10.45 | 79.73 | 8.2 |

Table S8. The photoelectric conversion efficiency of the used blue LED chip.

| Current (mA) | Voltage (V) | Output power (mW) | Photoelectric conversion efficiency (%) |
| --- | --- | --- | --- |
| 20 | 2.63 | 28.83 | 54.8 |
| 40 | 2.71 | 57.58 | 53.1 |
| 60 | 2.77 | 85.43 | 51.4 |
| 80 | 2.86 | 112.20 | 49.0 |
| 100 | 2.87 | 138.30 | 48.2 |
| 120 | 2.92 | 163.70 | 46.7 |
| 140 | 2.96 | 188.00 | 45.4 |

**Fig. S17 a** PL spectra of the pc-LED before (pristine) and after being placed in air for 6 months, measured under 100 mA. **b** Stability test of pc-LED with continuous operation time at 100 mA.

**References:**

1 Zhou, X. et al. An ultraviolet-visible and near-infrared-responded broadband NIR phosphor and its NIR spectroscopy application. *Adv. Opt. Mater.* **8**, 1902003 (2020).

2 De Guzman, G. N. A. et al. Near-infrared phosphors and their full potential: a review on practical applications and future perspectives. *J. Lumin.* **219**, 116944 (2020).

3 Zhao, F. Y. et al. Octahedron-dependent near-infrared luminescence in Cr^3+^-activated phosphors. *Mater. Today Chem.* **23**, 100704 (2022).

4 Mei, H. et al. Compositional design, structure stability, and microwave dielectric properties in Ca_3_MgBGe_3_O_12_ (B = Zr, Sn) garnet ceramics with tetravalent cations on B-site. *Ceram. Int.* **48**, 4658-4664 (2022).

5 Liu, H. et al*.* Structure, optical spectroscopy properties and thermochromism of Sm_3_Fe_5_O_12_ garnets. *J. Mater. Chem. C* **4**, 10529-10537 (2016).

6 Yao, L. et al. Simultaneous absorption and near-infrared emission enhancement of Cr^3+^ ions in MgGa_2_O_4_ spinel oxide via anionic F-substitution. *Adv. Opt. Mater.* 11, 2202458 ( 2023).

7 Yang, Z. et al. Giant red-shifted emission in (Sr,Ba)Y_2_O_4_:Eu^2+^ phosphor toward broadband near-infrared luminescence. *Adv. Funct. Mater.* **32**, 2103927 (2022).

8 Wei, Y. et al. Anti-thermal-quenching Bi^3+^ luminescence in a cyan-emitting Ba_2_ZnGe_2_O_7_:Bi phosphor based on zinc vacancy. *Laser Photonics Rev.* **15**, 2000048 (2020).

9 Li, X. et al. Study of oxygen vacancies' influence on the lattice parameter in ZnO thin film. *Mater. Lett.* **85**, 25-28 (2012).

10 Huang, D. et al. Cr,Yb-codoped Ca_2_LaHf_2_Al_3_O_12_ garnet phosphor: electronic structure, broadband NIR emission and energy transfer properties. *Dalton T.* **50**, 908-916 (2021).

11 Malysa, B., Meijerink, A. & Juestel, T. Temperature dependent Cr^3+^ photoluminescence in garnets of the type X_3_Sc_2_Ga_3_O_12_ (X = Lu, Y, Gd, La). *J. Lumin.* **202**, 523-531 (2018).

12 Jiang, L. et al. Ultra-broadband near-infrared Gd_3_MgScGa_2_SiO_12_:Cr, Yb phosphors: photoluminescence properties and LED applications. *J. Alloy. Compd*. **920**, 165912 (2022).

13 Dumesso, M. U. et al. Efficient, stable, and ultra-broadband near-infrared garnetphosphors for miniaturized optical applications. *Adv. Opt. Mater.* **10**, 2200676 (2022).

14 Liu, Y. et al. Broadband NIR garnet phosphors with improved thermal stability via energy transfer. *ACS Appl. Electron. Mater.*  **4**, 643-650 (2022).

15 He, S. et al. Efficient super broadband NIR Ca_2_LuZr_2_Al_3_O_12_:Cr^3+^,Yb^3+^ garnet phosphor for pc-LED light source toward NIR spectroscopy applications. *Adv. Opt. Mater.* **8**, 1901684 (2020).

16 Zhang, Q. et al. Enhancing and tuning broadband near-infrared (NIR) photoluminescence properties in Cr^3+^-doped Ca_2_YHf_2_Al_3_O_12_ garnet phosphors via Ce^3+^/Yb^3+^-codoping for LED applications. *J. Mater. Chem. C* **9**, 4815-4824 (2021).

17 Wang, Y. et al. Ultra-broadband and high efficiency near-infrared Gd_3_Zn*_x_*Ga_5-2_*_x_*Ge*_x_*O_12_:Cr^3+^ (*x* = 0–2.0) garnet phosphors via crystal field engineering. *Chem. Eng. J.* **437**, 135346 (2022).

18 Nie, W. et al. Cr^3+^-activated Na_3_X_2_Li_3_F_12_ (X = Al, Ga, or In) garnet phosphors with broadband NIR emission and high luminescence efficiency for potential biomedical application. *J. Mater. Chem. C* **9**, 15230-15241 (2021).

19 Li, C. et al. Efficient and thermally stable broadband near-infrared emission in a garnet Gd_3_In_2_Ga_3_O_12_:Cr^3+^ phosphor. *Dalton T.* **51**, 16757-16763 (2022).

20 Jia, Z. et al. Strategies to approach high performance in Cr^3+^-doped phosphors for high-power NIR-LED light sources. L*ight: Sci. Appl.* **9**, 86 (2020).

21 Mao, N. et al. A broadband near-infrared phosphor Ca_3_Y_2_Ge_3_O_12_:Cr^3+^ with garnet structure. *J. Alloy. Compd*. **863**, 158699 (2021).
